# Development and Validation of an Automated Tool to Retrieve and Curate Faculty Publications of Academic Departments

**DOI:** 10.7759/cureus.47976

**Published:** 2023-10-30

**Authors:** Richard H Epstein, Dorothee A Mueller, Jeremy P Walco, Carmen D Manresa, Shawn E Banks, Robert E Freundlich

**Affiliations:** 1 Anesthesiology, Perioperative Medicine and Pain Management, University of Miami Miller School of Medicine, Miami, USA; 2 Anesthesiology, Vanderbilt University Medical Center, Nashville, USA

**Keywords:** library science, scopus, pubmed, authorship, bibliometrics

## Abstract

Introduction

Academic departments need to monitor their faculty's academic productivity for various purposes, such as reporting to the medical school dean, assessing the allocation of non-clinical research time, evaluating for rank promotion, and reporting to the Accreditation Council for Graduate Medical Education (ACGME). Our objective was to develop and validate a simple method that automatically generates query strings to identify and process distinct department faculty publications listed in PubMed and Scopus.

Methods

We created a macro-enabled Excel workbook (Microsoft, Redmond, WA) to automate the retrieval of faculty publications from the PubMed and Scopus bibliometric databases (available at https://bit.ly/get-pubs). Where the returned reference includes the digital object identifier (doi), a link is provided in the workbook. Duplicate publications are removed automatically, and false attributions are managed.

Results

At the University of Miami, between 2020 and 2021, there were 143 anesthesiology faculty-authored publications with a PubMed identifier (PMID), 95.8% identified by the query and 4.2% missed. At Vanderbilt University Medical Center, between 2019 and 2021, there were 760 anesthesiology faculty-authored publications with a PMID, 94.3% identified by the query and 5.7% missed. Recall, precision, and the F1 score were all above 93% at both medical centers.

Conclusions

We developed a highly accurate, simple, transportable, scalable method to identify publications in PubMed and Scopus authored by anesthesiology faculty. Manual checking and faculty feedback are required because not all names can be disambiguated, and some references are missed. This process can greatly reduce the burden of curating a list of faculty publications. The methodology applies to other academic departments that track faculty publications.

## Introduction

Despite controversy about the appropriate weighting of research and publication, teaching, and service related to academic review, promotion, and tenure, authorship remains an important criterion [1]. Academic departments continually monitor their faculty's academic productivity for institutional reporting, assessing if non-clinical research time is being used productively, and reporting to the Accreditation Council for Graduate Medical Education. Typically, this is a labor-intensive process incorporating manual searching of publication databases and repeated requests to faculty to provide their publications [2]. Improving the efficiency of such tasks is, thus, of interest. Identifying faculty publications is non-trivial because of challenges related to name disambiguation (i.e., distinguishing among distinct authors who share the same name), alternative representations of authors' names, and inconsistent listing of departmental and institutional affiliations.

In this study, we developed and validated a macro-enabled Excel workbook (Microsoft, Redmond, WA) that automatically generates query strings to identify and process distinct department faculty publications listed in PubMed (National Library of Medicine) or Scopus (Elsevier). Scopus includes some journals not indexed in PubMed and all articles with a PMID, but there is often a delay until such articles appear in Scopus. We did not use Google Scholar (Alphabet, Mountain View, CA) because of search string length limitations (256 characters), nonsupport of complex search expressions, absence of search export functions, and potential blocking issues related to multiple searches from a single IP address [3]. We also excluded Web of Science (Clarivate, London, UK) because constraining searches by department affiliation is not supported, a critical step to limit the number of false attributions.

## Materials and methods

The institutional review boards at the University of Miami (UM) and Vanderbilt University Medical Center (VUMC) determined that this study is non-human subject research (July 25, 2022, and August 21, 2022, respectively).

Basic strategy

The basic strategy to identify faculty publications with a PMID was based on the year of electronic publication and the names of the faculty, department, and institution. The goal was to achieve high sensitivity at the cost of somewhat lower specificity because of the greater importance of not missing publications. As is the case for all automated processes identifying authorship, manual confirmation of identified publications is required, and faculty need to provide feedback to identify missing publications (e.g., not indexed in either PubMed or Scopus).

Development of the publication search criteria

We first identified how the department and institution were listed in PubMed, expecting some variability. At UM, an examination of a convenience sample of departmental faculty publications revealed that the affiliated department (AD) fields overwhelmingly included the string "Department of Anesthesiology" and, occasionally, "Department of Anesthesia," but in some cases, no department was listed. The vast majority of AD fields also included the string "University of Miami," but sometimes only "Miller School of Medicine." These four terms were, thus, used for the automated creation of the search strings using a combination of Excel formulas and Visual Basic for Applications (Microsoft).

At VUMC, the related AD terms were "Vanderbilt University" or "Vanderbilt Medical Center" or "Monroe Carell" as the institution (their children's hospital) and "Anesthesia" or "Anesthesiology" as the department.

Identifying faculty members

The second step in the process was generating the department faculty's names at each institution and determining variations in the bibliographic database. We identified several individuals who published under different versions of their names or whose names changed due to marriage. Listing a middle initial was inconsistent, so only the first initial and last name were used. Multiple entries were made for faculty who had used different names, with a unique identifier assigned to the faculty member, and applied to each alternative name, so they could be linked. We could not use the Open Researcher and Contributor ID (ORCID) to reliably identify authors because many faculty did not have a publicly accessible ORCID, and many journals do not require that authors provide this identifier.

Identifying publications where the department was missing

For the searches that included the faculty name, department, and institution, the entire PubMed and Scopus publication databases were searched, with no restriction on the included journals. There were occasional faculty publications where the organization was listed but not the department. Thus, we added to the search string the organizational names and faculty names without a department criterion but limited the search to anesthesia, critical care, and pain management journals, as identified from the full titles of the 35,048 journals indexed in PubMed at the time of the analysis. We also included some additional journals not including the search strings (i.e., anesth, anaesth, critical care, intensive care, pain, regional) where faculty published (e.g., Journal of Medical Systems, Perioperative Care and Operating Room Management). All PubMed journals were not included in this supplemental search because the number of false attributions would have been excessive due to author name similarities.

Building the Excel workbook

**Figure 1:**
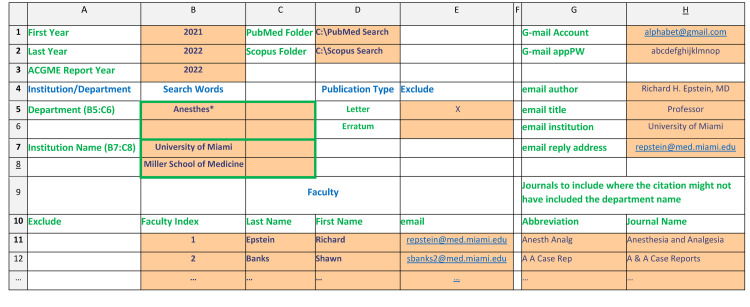
Setup fields for the publication retrieval workbook The columns and rows correspond to the Excel setup worksheet. The fields with orange highlighting contain the input used to create the search query, including the date range, the location of the folders where the output of the search results are to be placed, the keywords used to identify the institution and department, and whether letters or erratum are to be excluded from the search. Each faculty member's last and first name is entered in columns C and D, respectively, and a unique index is applied to each. The initial of the first name is calculated automatically. If a faculty has more than one representation of their name, the same index is used for each entry. Journal abbreviations and full names for specialty journals in which departmental faculty publish but where the department name is occasionally omitted or as a matter of journal policy are also entered. A G-mail account and password must be provided to use the automated email macro in the workbook. The underlining of the hyperlinked email addresses is generated automatically by Excel.

A macro-enabled Excel workbook (Microsoft, Redmond, WA) was created using Excel formulas and Visual Basic for Applications (VBA) to automate the construction of the queries and processing of the output from the PubMed and Scopus searches. This workbook is provided at https://bit.ly/get-pubs and includes detailed instructions. The user is guided through the sequential process of clicking the macro buttons. The PubMed and Scopus queries are constructed based on user-supplied parameters (Figure 1) and copied into the search boxes at the respective internet sites (www.pubmed.gov and www.scopus.org). Duplicate publications are removed, citation strings are generated, and links to the digital object identifier (DOI) reference are added if provided. If needed to manually confirm an attribution, clicking the link displays the abstract and its metadata, including full author names and affiliations. False attributions are easily flagged in the workbook, resulting in their being moved to a separate worksheet and eliminated from subsequent searches or reporting. The names and number of departmental faculty authors are listed for each publication.

Confirming departmental publications with a PubMed Identifier (PMID)

At UM, each reference with a PMID first published between 2020 and 2021 was manually checked to confirm faculty authorship. Each faculty was sent the list of their publications for confirmation and to identify any additional publications that might have been missed. In addition, we searched for all publications in which the AD field included "University of Miami" or "Miller School of Medicine" published in 2020 or 2021 without author or department constraints and manually identified any that included at least one Department of Anesthesiology faculty member. Merging these searches provided the reference list of anesthesia department faculty publications for validation.

At VUMC, there was already a manual process curated by administrative personnel incorporating faculty feedback to track academic productivity. Because VUMC faculty receive financial compensation for publications, we expected that nearly all publications with a PMID would have been identified. Nonetheless, the automated process discovered a few absent references, which were added to create a reference list of VUMC faculty publications for validation.

Evaluation of algorithm performance for publications with a PMID

The workbook was used to confirm the attribution of each distinct publication with a PMID. These were compared to the reference lists of publications with a PMID at the two institutions. True positives (TP) were publications correctly attributed to a faculty member. False positives (FP) were publications incorrectly attributed to a faculty member. False negatives were publications with a PMID that the faculty had authored but were not identified by the algorithm. We computed the recall (TP/(TP+FN)), precision (TP/(TP+FP)), and the F1 score (2*TP/*(2*TP+FP+FN)) of the algorithm. We did not compute the accuracy ((TP+TN)/(TP+TN+FP+FN)) because that value is not meaningful, given the enormous number of true negatives (3,194,887 between 2020 and 2021 and 4,424,642 between 2019 and 2021). If computed, accuracy would have been nearly 100%, regardless of algorithm performance [4].

## Results

At UM, between 2020 and 2021, there were 143 distinct, validated publications with a PMID and at least one departmental faculty (Table 1). Of those 143, 95.8% were identified by the query, and 4.2% were missed. Recall, precision, and the F1 score were all above 93% (Table 1).

**Table 1:** Confirmation of search results for publications with a PubMed identifier at the studied departments Abbreviations: PMID, PubMed Identifier; UM, University of Miami; VUMC, Vanderbilt University Medical Center ^a^ Confirmed publications were those with a PMID where the author was a faculty member at the institution during the year of publication and verified manually (see Methods). The 760 publications at VUMC excluded 433 publications without a PMID, 18 that were published when the author was a faculty at a different institution, and 20 where the faculty was listed only as a collaborator, not an author, on a large, multicenter study. The confirmed list at UM only included references with a PMID.

Metric	UM	VUMC
Year of Publication	2020-2021	2019-2021
Confirmed publications^a^ (n)	143	760
References identified by the queries (n, %)	137 (95.8%)	717 (94.3%)
References missed by the queries (n, %)	6 (4.2%)	43 (5.7%)
Recall (sensitivity)	96.0%	93.4%
Precision	93.5%	96.2%
F1 score	94.7%	94.8%

At VUMC, between 2019 and 2021, there were 760 distinct, validated publications with a PMID on which there was at least one departmental faculty (Table 1). Of those, the query identified 94.3% and missed 5.7%. Similar to the results at UM, recall, precision, and the F1 score were all above 93% (Table 1).

Reasons why some publications were missed are presented in Table 2. The most common cause at both institutions was that the author's department was not specified, and the journal was not included in the list of journals to search when the department and or institution was missing from the citation.

**Table 2:** Reasons why faculty publications with a PubMed identifier were missed Abbreviations: UM, University of Miami; VUMC, Vanderbilt University Medical Center ^a^ Journals in which anesthesiologists often publish were searched without constraining the search to include the department as an affiliation. Other journals in which faculty published with a missing department affiliation were subsequently added to the list, which increased the sensitivity in Table 1 to 100% at UM and 98.3% at VUMC.

Reason	UM (n=6)	VUMC (n=43)
Department not specified and not in a listed journal^a^	6	30
Institution and department not specified	0	10
Institutional or author name misspelled	0	3

## Discussion

Our workbook performed well at identifying publications with a PMID authored by all faculty while they were department members and only missed a small percentage of relevant publications. Typically, missing manuscripts omitted the name of the department. Adding additional journals to search when the department is missing improved sensitivity. If the tracking of residents' and fellows' publications is desired, those names can easily be added. Although designed originally for bulk processing of all faculty publications, individuals can also use the workbook to track their publications or identify articles inadvertently omitted from their curriculum vitae. The query string for PubMed, provided on one of the worksheets, can be used to create a really simple syndication (RSS) feed from the PubMed website to identify the most recent faculty publications. Such an RSS is implemented at UM and runs weekly, with distribution to the department chair and vice-chair for faculty development. The workbook (https://bit.ly/get-pubs) is provided online for public use.

Manually checking attributed publications is important because name disambiguation cannot be completely achieved. Having a link to the article's DOI facilitates that process. We strongly recommend that faculty be sent a list of their identified publications for edits and additions. They also should be informed that book chapters and other non-indexed publications are not captured and that they will need to supply those manually. Such omissions are not a limitation of the algorithm, per se, but rather of the data sources. Once initially configured, we think the process is straightforward enough for continued use by an administrative assistant since only the list of faculty names and journal names needs to be curated and attributions confirmed. Missing publications can be manually entered following the format specified in the workbook.

Other investigators have addressed issues related to the ambiguity of author names in bibliographic databases, with some features similar to our method. For example, Torvik and Smalheiser developed a tool ("Author-ity") to estimate the probability that two articles in MEDLINE sharing the same author name were by the same person [5]. Their approach was based on multiple dimensions after removing stop words and some preprocessing, including author name features and variant spellings, email addresses, correlations between author last names and affiliation words, journal name, publication language, number of shared co-authors, number of shared title words, number of shared affiliation words, and number of shared MeSH words. However, their online tool only covers MEDLINE articles published through September 2008 [6] and is limited to one author at a time. Liu et al. improved on the Torvik and Smalheiser methodology for disambiguating authors in PubMed using an unsupervised machine learning approach based on agglomerative clustering using consideration of the title, affiliation, grant information, journal, abstract, substance, Mesh words, author information, and publication date [7]. Their algorithm is the current method that PubMed uses to return author publications when one clicks on an author's name in a reference [7]. Similar to the Torvik and Smalheiser methodology, this process is not designed for batch processing. However, what the algorithm in the current paper provides for PubMed searches, functionally, is what is returned from the Liu et al. methodology for all listed authors in the department, but further constrained by the department and institutional affiliation and the year of publication, further eliminating publications already identified from previous queries.

If all journals required that authors supply an ORCID or some similar universal identifier such as the Web of Science ResearcherID [8] or International Standard Name Identifier (ISNI) [9], future problems related to name disambiguation would be greatly mitigated. For example, if supplied by the journal, PubMed searches return the ORCID in the AUID field. However, such requirements are not currently in place, many authors do not have one of these identifiers, and most historical publications would not have this information.

Limitations

The algorithms were evaluated at two anesthesia departments, and despite similar performance, results may not be fully generalizable. Nonetheless, we suspect that the approach will translate well to other medical specialties. However, for basic science disciplines where widespread collaborative studies are common (e.g., cell biology), the hundreds to thousands of authors on some papers will likely increase the number of false attributions. Moreover, manual reconciliation would be necessary for departments where some faculty share the same first initial and last name. Finally, a limitation exists for Scopus searches in that, if more than 2,000 references are identified, a link is sent to the list rather than downloading the file and that online link did not include the PMID at the time of the study. However, that issue would likely only arise for a department if the search spanned many years of publication history and can easily be addressed by incrementally uploading sequential search year ranges. To use the embedded email system, a G-mail account is required. However, those are available without charge and have the advantage of being able to use a password separate from the account's main password.

## Conclusions

We developed and validated an accurate, transportable, scalable method using a macro-enabled Excel workbook to identify publications in PubMed and Scopus authored by anesthesia department faculty. The process removes duplicates, easily excludes incorrect attributions, provides publications' DOI links, and allows email distribution to faculty for confirmation. The described process should reduce the required effort to retrieve and curate indexed faculty publications. The workbook can also be used by individuals to decrease the effort required to keep their curriculum vitae current.
